# TGF-β Signalling Mediates the Anti-Inflammatory Activity of Enamel Matrix Derivative In Vitro

**DOI:** 10.3390/ijms23179778

**Published:** 2022-08-29

**Authors:** Layla Panahipour, Mariane Beatriz Sordi, Zahra Kargarpour, Reinhard Gruber

**Affiliations:** 1 Department of Oral Biology, University Clinic of Dentistry, Medical University of Vienna, Sensengasse 2a, 1090 Vienna, Austria; 2 Department of Periodontology, School of Dental Medicine, University of Bern, Freiburgstrasse 7, 3010 Bern, Switzerland; 3 Austrian Cluster for Tissue Regeneration, Donaueschingenstraße 13, 1200 Vienna, Austria

**Keywords:** enamel matrix derivative, TGF-β, inflammation, macrophages, periodontal regeneration

## Abstract

Enamel matrix derivative (EMD) prepared from extracted porcine fetal tooth material can support the regrow of periodontal tissues. Previous findings suggest that EMD has anti-inflammatory properties and TGF-β activity in vitro. However, the anti-inflammatory activity of EMD is mediated via TGF-β has not been considered. To this aim, we first established a bioassay to confirm the anti-inflammatory activity of EMD. The bioassay was based on the RAW 264.7 macrophage cell line and proven with primary macrophages where EMD significantly reduced the forced expression of IL-6. We then confirmed the presence of TGF-β1 in EMD by immunoassay and by provoking the Smad2/3 nuclear translocation in RAW 264.7 macrophages. Next, we took advantage of the TGF-β receptor type I kinase-inhibitor SB431542 to block the respective signalling pathway. SB431542 reversed the anti-inflammatory activity of EMD and TGF-β in a bioassay when IL-6 and CXCL2 expression was driven by the LPS stimulation of RAW 264.7 macrophages. This central observation was supported by showing that SB431542 reversed the anti-inflammatory activity of EMD using IL-1β and TNF-α-stimulated ST2 bone marrow stromal cells. Together, these findings implicate that the TGF-β activity mediates at least part of the anti-inflammatory activity of EMD in vitro.

## 1. Introduction

Periodontal disease is an inflammatory disorder that drives catabolic cellular events, ultimately leading to tooth loss due to a lack of supporting tissues [1,2,3]. It is fundamental to understand the pathological mechanisms on a cellular and molecular basis to implement therapies aiming to modulate inflammation and allow for regenerative strategies. [2,3] In this sense, enamel matrix derivative (EMD) has been indicated in approaches for periodontal regeneration [4,5,6,7]. In vitro, EMD has proven anti-inflammatory properties [8,9,10,11]. For instance, EMD reduced the LPS-induced TNF-α production in rat primary monocytes [8]. EMD diminished the pro-inflammatory activity and increased the expression of tissue repair mediators, such as vascular endothelial growth factor, in monocyte-derived macrophages challenged by LPS [11]. In human blood-derived cells stimulated with LPS, EMD depressed TNF-α release [9]. In LPS-exposed human osteogenic cells and human epithelial gingival keratinocytes, EMD decreased the expression of inflammatory cytokines [10]. Even with the vastly proven in vitro anti-inflammatory properties of EMD and considering that EMD is a device containing several active components, it still remains unclear what is causing such reduction in pro-inflammatory activity.

EMD is an extract of the enamel matrix from the tooth germ of piglets. Proteome analyses have confirmed the presence of enamel matrix proteins, namely amelogenin and ameloblastin [12], and growth factors such as TGF-β in the EMD composition [13,14]. Amelogenin is raised as the main component of EMD [11]. Amelogenins represent a family of proteins involved in the formation of the enamel matrix, before the initiation of enamel biomineralization, and constitute up to 90% of the enamel matrix [4,15]. The amelogenin gene is a tooth-specific gene expressed in pre-ameloblasts, ameloblasts, and epithelial root sheath remnants [16,17]. In vitro, amelogenin reprogrammed U937 macrophages and CD14^+^ peripheral blood-derived monocytes-derived macrophages into the anti-inflammatory M2 phenotype [18]. This knowledge, however, is well known, while information that the anti-inflammatory activity of EMD could be mediated via TGF-β has not been yet considered.

There is indirect evidence that EMD has a potent TGF-β activity, which is attributed to SB431542, a pharmacologic inhibitor of the TGF-β type I receptor that blocked the effects of EMD in vitro. For instance, SB431542 blocked all genes being increasingly expressed when palatal fibroblasts were exposed to EMD [19]. Furthermore, SB431542 reversed the inhibitory effect of EMD on adipogenic differentiation, [13] and abolished the effect of EMD on osteoclastogenesis [20]. There is thus evidence for a TGF-β activity of EMD—and also good reason to suggest that the anti-inflammatory activity of EMD [8,9,10] is caused by TGF-β activity.

It is widely established that TGF-β has a strong anti-inflammatory activity in macrophages [21,22,23,24]. In RAW 264.7 macrophage-like cells and primary macrophages, recombinant TGF-β1 inhibited the LPS-induced expression of various inflammatory mediators, including IL-6 and CXCL2 [22]. Therefore, the present study aimed to test the hypothesis that the anti-inflammatory effects of EMD are caused by intrinsic TGF-β activity. To test this premise, we took advantage of our established in vitro bioassay where RAW 264.7 macrophages exposed to LPS respond with an increased expression of IL-6 and CXCL2. We have used this bioassay to show the anti-inflammatory activity of PRF, for instance [25]. Herein, we confirm the anti-inflammatory activity of EMD and extend this observation, highlighting TGF-β signalling as the potential route of anti-inflammatory responses on macrophages in vitro.

## 2. Results

### 2.1. EMD Reduces Saliva-Induced IL-6 Expression and Protein Release in RAW 264.7 Cells and Primary Macrophages

**Figure 1:**
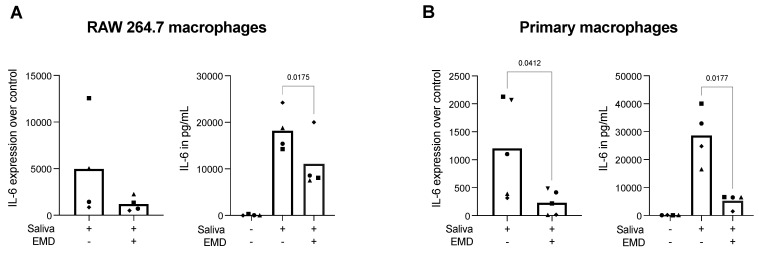
Gene expression of IL-6 and IL-6 immunoassay in RAW 264.7 cells (**A**) and primary macrophages (**B**) under 5% saliva stimulation. EMD diminished the elevated IL-6 expression and secretion after saliva challenge in RAW 264.7 cells and primary macrophages. Each data point represents an independent experiment. To compare groups, paired *t*-tests were applied, and the statistically significant *p* values were added to graphs.

Although this study aimed to evaluate if the anti-inflammatory effects of EMD could be mediated via TGF-β activity, we first sought to confirm the anti-inflammatory potential of EMD in macrophages stimulated with saliva, knowing that the effects are mediated via the TLR4 [26,27]. After a dose–response of EMD in RAW 264.7 cells to verify the cell viability, we determined that 100 µg/mL of EMD provides a safe appliance of the material on a macrophage cell line (Supplementary Figure S1). Then, we challenged RAW 264.7 cells and primary macrophages obtained from mice bone marrow with 5% saliva [26], which led to an elevated expression of IL-6. In this setting, EMD significantly reduced IL-6 at the protein level in both RAW 264.7 cells and primary macrophages, and diminished IL-6 at the transcriptional level in primary macrophages, also presenting a trend in reducing IL-6 expression in RAW 264.7 cells (Figure 1). These data confirmed that the in vitro bioassay on macrophages works well and that EMD can reduce inflammation in RAW 264.7 cells and primary macrophages.

### 2.2. RAW 264.7 Cells Treated with EMD or rTGF-β1 Contains TGF-β in Its Supernatants

**Figure 2:**
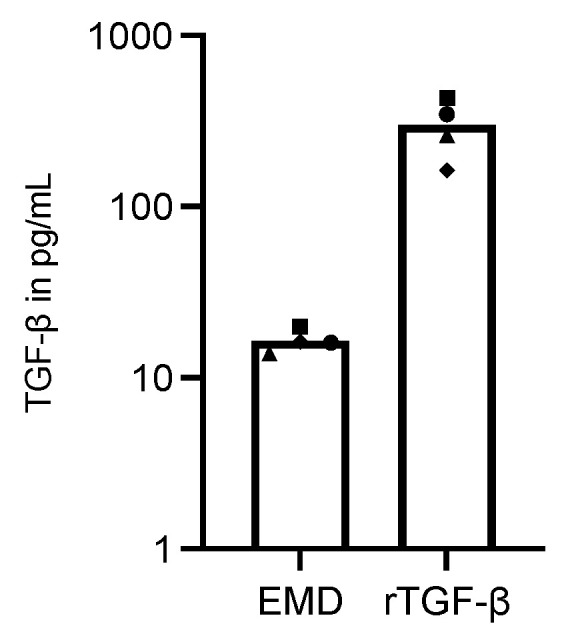
TGF-β immunoassay in RAW 264.7 macrophages treated with EMD or recombinant TGF-β. EMD induced TGF-β production in stimulated RAW 264.7 cells. Each data point represents an independent experiment.

Then, to check on the protein levels of TGF-β in the supernatant of RAW 264.7 cells stimulated with EMD or rTGF-β1, we performed immunoassay analyses. We found out that, even though the rTGF-β1 treated cells have shown an extremely high quantity of TGF-β—as expected, considering we are applying TGF-β itself—EMD treated cells showed TGF-β protein levels in the range of 17 pg/mL (Figure 2), confirming that TGF-β is present in the supernatant of EMD-primed RAW 264.7 cells.

### 2.3. EMD Reduces Inflammation in LPS-Stimulated RAW 264.7 Cells, Which Is Reversed by SB431542

**Figure 3:**
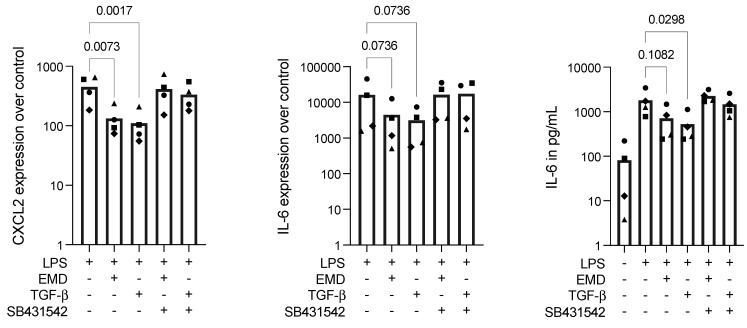
Gene expression of CXCL2 and IL-6 and IL-6 protein secretion in RAW 264.7 cells under LPS stimulation. EMD and TGF-β significantly reduced CXCL2 expression and showed a trend in reducing IL-6 expression and secretion in LPS-stimulated RAW 264.7 cells. SB431542 reversed the reduced effect provoked by EMD and TGF-β. Each data point represents an independent experiment. To compare groups, Friedman test followed by Dunn’s multiple comparison tests on the relevant groups were applied.

Next, we introduced LPS instead of saliva; both act on the same pathway of TLR4 [26,27]. Consequently, after stimulation with LPS, RAW 264.7 cells expressed high CXCL2 and IL-6, which was reduced by applying EMD or TGF-β, as expected. Then, to assess if the EMD activity on the reduction of inflammatory activity in macrophages was mediated by TGF-β, we introduced the TGF-β receptor type I kinase-inhibitor SB431542. SB431542 overturned the reduced expression of the inflammatory markers by EMD and TGFβ, bringing the expressions of CXCL2 and IL-6 to similar levels of the LPS stimulation alone (Figure 3). Furthermore, we performed the LPS stimulation in EMD-primed primary macrophages, which were also blocked with the SB431542; also, in this case, LPS provoked high expression levels of IL-6, which were reduced with the application of EMD and further increased in the presence of SB431542 (Table 1). There is, thus, an indication that the EMD action on anti-inflammatory activity in LPS-challenged macrophages is mediated by TGF-β.

### 2.4. EMD Attenuates the Nuclear Translocation of Smad2/3 in RAW 264.7 Macrophages

**Figure 4:**
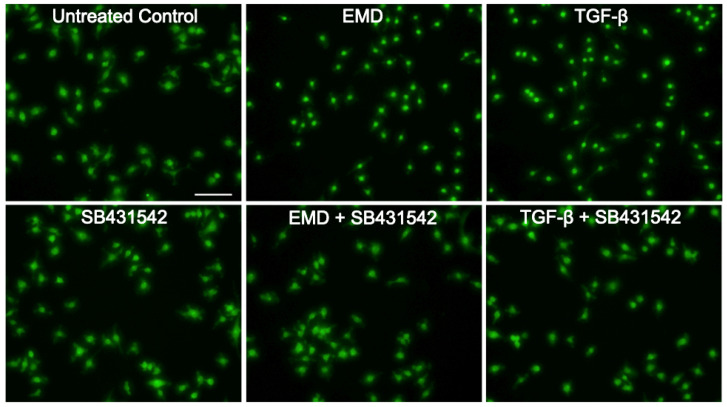
Smad2/3 immunofluorescence in RAW 264.7 cells. EMD and TGF-β attenuated the nuclear translocation of Smad2/3. Thepositive cells show a focused signal indicating the nuclear translocation of the Smad2/3 immunofluorescence. This effect was reversed with the application of SB431542, hence, the signal is more dispersed throughout the cytoplasm. SB431542 alone did not provoke changes in the nuclear translocation of Smad2/3. The scale bar represents 100 µm.

Considering that TGF-β stimulates Smad2/3 phosphorylation, [28] we sought to evaluate if EMD could activate the TGF-β-related Smad2/3 signalling pathway. Then, we stimulated RAW 264.7 cells with EMD or TGF-β, in combination or not with the SB431542. SB431542 alone was also applied as a control. Immunofluorescence images revealed that EMD and TGF-β attenuated the nuclear translocation of Smad2/3, and this effect was reversed in the presence of SB431542 (Figure 4). This is further indication that EMD’s anti-inflammatory properties are mediated via TGF-β signalling.

### 2.5. EMD Shows a Trend in Diminishing Inflammation in IL-1β+TNF-α-Stimulated ST2 Cells

**Figure 5:**
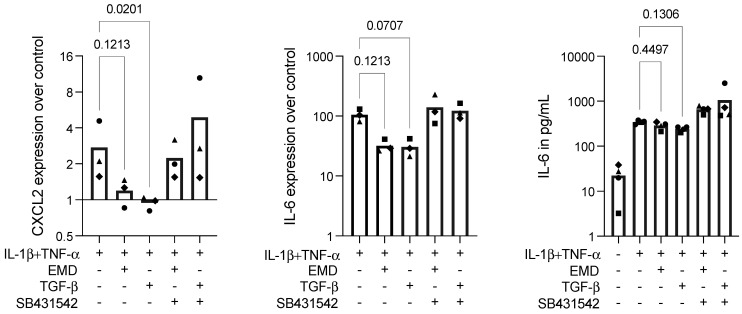
Gene expression of CXCL2 and IL-6, and IL-6 protein secretion in ST2 cells under IL-1β+TNF-α stimulation. TGF-β significantly reduced CXCL2 expression. EMD and TGF-β showed a trend in reducing CXCL2 and IL-6 at the transcriptional level, which was reversed by applying SB431542 in IL-1β+TNF-α-stimulated ST2 cells. The protein levels of IL-6 in IL-1β+TNF-α-stimulated ST2 cells were not significantly reduced by EMD or TGF-β. Each data point represents an independent experiment. To compare groups, the Friedman test followed by Dunn’s multiple comparison tests on the relevant groups were applied.

To confirm the data obtained by applying the bioassay on macrophages, we introduced IL-1β+TNF-α inflammatory stimulation in ST2 mesenchymal cells. After stimulation, ST2 cells expressed high levels of IL-6 and some expression of CXCL2, which were reduced in the presence of EMD or TGF-β, as expected. Again, to assess if the EMD activity on the reduction of inflammatory activity in ST2 cells was mediated by TGF-β, we added SB431542 to this setting. SB431542 overturned the expression of the inflammatory markers provoked by EMD and TGF-β, increasing the expression levels of IL-6 and CXCL2 (Figure 5). Hence, there is an indication that the EMD’s action on anti-inflammatory activity in ST2 mesenchymal cells is also mediated by TGF-β.

## 3. Discussion

EMD was introduced to support periodontal regeneration clinically back in 1997 [29] and has led to numerous preclinical studies trying to decipher the molecular and cellular mechanism of its activity [8,9,13,14,19,20,30]. Since amelogenins consist of 90% of the total protein content of EMD, [4] most of the EMD beneficial activity is attributed to the amelogenins. Amelogenins are self-assembling extracellular matrix proteins required for enamel development that can bind to cells via membrane proteins, such as the lysosomal membrane proteins, the most investigated binding proteins of amelogenins [31]. However, even though recombinant amelogenin is suggested to regenerate periodontal tissues, [32] there is no obvious impact on controlling an inflammatory response in vitro. The well-supported anti-inflammatory activity of EMD can thus not be explained by the highly abundant amelogenins. In this sense, the main finding of the present study is that it is the TGF-β activity of EMD that accounts for the robust anti-inflammatory activity of EMD.

This conclusion is mainly based on the observation that the TGF-β receptor type I kinase-inhibitor, namely SB431542, blocks the TGF-β activity of EMD. In the presence of SB431542, EMD failed to exert its anti-inflammatory activity. These findings agree with previous studies on the role of EMD-derived TGF-β in changing gene expression on palatal fibroblasts, [19] to suppress adipogenesis, [13] and to reduce osteoclastogenesis [20]. Our findings that EMD pushed Smad2/3 nuclear translocation in RAW 264.7 macrophages support the observations with other cell types, for example, when EMD stimulated the phosphorylation and nuclear accumulation of Smad2 in oral epithelial and fibroblastic human cells [33] and increased the expression of Smad3 in primary human osteoblasts. [34] Also consistent with other studies is that we have identified TGF-β by immunoassay, thus adding to the accumulating evidence as previously reported [30,35,36]. In addition, our data also confirm the strong pro-inflammatory activity of saliva [26,37,38,39], which serves as a model to confirm the anti-inflammatory activity of EMD in murine RAW 264.7 cells and primary macrophages.

The clinical relevance of our findings remains at a level of speculation. Nevertheless, there is indirect support for the anti-inflammatory activity of EMD also in vivo. Experimental periodontitis in rats was created by elevating a full-thickness gingival flap and ligating silk threads around the mandible first molars; the expression of IL-1β was significantly decreased after 14 days of treatment with EMD when compared to control animals [40]. Furthermore, EMD treatment reduced the expression of IL-8 and TNF-α in the human whole blood [9] and the expression of MMP-1 in the human crevicular fluid [41]. Immunoassays on the protein level of TGF-β in human periodontal ligament cells treated with 50 µg/mL of EMD for 6 h reached about 7 pg/mL [35], while herein, we report 17 pg/mL of TGF-β in RAW 264.7 cells treated with 100 µg/mL of EMD for 6 h. There were also immunoassays confirming TGF-β1 in EMD, thus not produced by any cells in a bioassay [13]. These data further support the notion that the anti-inflammatory activity of EMD results from the endogenous TGF-β.

## 4. Materials and Methods

### 4.1. Cell Lineages

Primary macrophages were obtained from BALB/c mice at the age of 6-8 weeks purchased from Animal Research Laboratories, Himberg, Austria. The femora and tibiae of the mice were removed after scarifying, and bone marrow cells were collected. Bone marrow cells were seeded at 1 × 10^6^ cells/cm^2^ into 24-well plates and grown for 7 days in Dulbecco’s Modified Eagle Medium (DMEM; Sigma Aldrich, St. Louis, MO, USA) supplemented with 10% fetal calf serum (FCS; Capricorn Scientific GmbH, Ebsdorfergrund, Germany), 1% of 10,000 units penicillin and 10 mg/mL streptomycin (PS; Sigma, St Louis, MO, USA), and with 20 ng/mL mouse macrophage colony-stimulating factor (M-CSF; ProSpec-Tany TechnoGene Ltd., Rehovot, Israel). RAW 264.7 macrophage-like cells (ATCC; LGC Standards GmbH, Wesel, Germany) were seeded at 3 × 10^5^ cells/cm^2^ into 24-well plates. ST2 murine mesenchymal cells were isolated from mouse bone marrow (Riken Cell Bank, Tsukuba, Japan) and seeded at 3 × 10^5^ cells/cm^2^ into 24-well plates.

### 4.2. Cell Stimulation

Cells were primed with 100 µg/mL of enamel derivative matrix (EMD; Straumann AG, Basel, Switzerland) for 1 h and then exposed to 100 ng/mL of LPS from *Escherichia coli* 055:B5 (Sigma Aldrich, St. Louis, MO, USA) for 6 h to induce an inflammatory response. Alternatively, 5% saliva [26] or 20 ng/mL IL-1β (ProSpec, Ness-Ziona, Israel) and TNF-α (ProSpec, Ness-Ziona, Israel) were used for cell stimulation. In parallel, cells were exposed to 10 ng/mL of recombinant human TGF-β1 (ProSpec, Ness-Ziona, Israel) for 1 h before the inflammatory stimulation. Alternatively, 10 µM of SB431542 (Calbiochem, Merck Millipore, Burlington, MA, USA), a TGF-β receptor I kinase inhibitor, was used together with EMD or TGF-β.

### 4.3. Real-Time Polymerase Chain Reaction (RT-PCR) and Immunoassay

For RT-qPCR, after stimulation, total RNA was isolated with the ExtractMe total RNA kit (Blirt S.A., Gdańsk, Poland), followed by reverse transcription and polymerase chain reaction (LabQ, Labconsulting, Vienna, Austria) on a CFX Connect™ Real-Time PCR Detection System (Bio-Rad Laboratories, Hercules, CA, USA). The mRNA levels were calculated by normalizing to the housekeeping gene GAPDH using the ^ΔΔ^Ct method. Primer sequences were CXCL2-F: CATCCAGAGCTTGAGTGTGACG, CXCL2-R: GGCTTCAGGGTCAAGGCAAAC, IL-6-F: GCTACCAAACTGGATATAATCAGGA, IL-6-R: CCAGGTAGCTATGGTACTCCAGAA, GAPDH-F: AACTTTGGCATTGTGGAAGG, GAPDH-R: GGATGCAGGGATGATGTTCT. For the immunoassays, the mouse IL-6 kit was used for RAW 264.7 cells stimulated with saliva or LPS, and the TGF-β kit was used to evaluate the quantity of TGF-β in the supernatant of EMD-primed or TGF-β-primed RAW 264.7 cells. The immunoassay kits were used according to the manufacturer’s instructions (R&D Systems, Minneapolis, MN, USA).

### 4.4. Immunofluorescence Analysis

Immunofluorescence analysis of Smad2/3 nuclear translocation was performed in RAW 264.7 cells. The cells were plated on Millicell^®^ EZ slides (Merck KGaA, Darmstadt, Germany) at a density of 3 × 10^4^ cells/cm^2^. Cells were stimulated with EMD or TGF-β, with and without the SB431542 inhibitor. Cells were fixed with 4% paraformaldehyde, blocked with 1% bovine serum albumin (BSA; Sigma-Aldrich, St. Louis, MO, USA) and permeabilized with 0.3% Triton X-100 (Sigma-Aldrich, St. Louis, MO, USA). Smad2/3 (1:800; D7G7 XP^®^, Cell Signaling, Danvers, MA, USA, #8685) was applied to the fixed cells overnight at 4 °C. Detection was performed with a goat anti-rabbit Alexa 488 secondary antibody (CS-4412, 1:800, Cell Signaling Technology). We captured the images on a fluorescence microscope with the DAPI-FITC dual excitation filter block (Echo Revolve Fluorescence Microscope, San Diego, CA, USA).

### 4.5. Statistical Analysis

All experiments were performed at least three times. Statistical analyses were performed with paired *t*-tests or the Friedmann test followed by Dunn’s multiple comparison test whenever appropriate. Analyses were performed using Prism v.9 (GraphPad Software; San Diego, CA, USA). Significance was set at *p* < 0.05.

## 5. Conclusions

With the inherent limitations of an in vitro study, we conclude by corroborating the already known anti-inflammatory activity of EMD in vitro [8,9,10,11] and extend this observation by highlighting that TGF-β signalling is the potential route of the anti-inflammatory property of EMD on macrophages. Nevertheless, further speculation is necessary to confirm our data. For instance, here, we applied murine macrophages and mesenchymal cell lines. In contrast, human periodontal-related cells might be challenged with TLR4 agonists (i.e., LPS, saliva) and treated with EMD and TGF-β to assess the anti-inflammatory potential of EMD and related the results with those of TGF-β. Further in-depth studies on the composition and the proportion of the components of EMD may respond to unanswered questions regarding the anti-inflammatory signalling pathway that EMD exerts on cells and tissues.

## Figures and Tables

**Table 1:** Gene expression of IL-6 (fold expression over control) in primary macrophages under LPS stimulation. EMD reduced IL-6 expression provoked by LPS stimulation in primary macrophages. SB431542 reversed the effect provoked by EMD, bringing up the IL-6 expression levels.

LPS	EMD + LPS	SB431542 + EMD + LPS
2128	54	1016
2067	284	4753

## Data Availability

All raw data are made available on request.

## Supplementary Materials

The supporting information can be downloaded at: https://www.mdpi.com/article/10.3390/ijms23179778/s1.

## References

[1] Schwarz F., Derks J., Monje A., Wang H.L. (2018). Peri-implantitis. J. Periodontol..

[2] Kinane D.F., Stathopoulou P.G., Papapanou P.N. (2017). Periodontal diseases. Nat. Rev. Dis. Primers.

[3] Kajiya M., Kurihara H. (2021). Molecular Mechanisms of Periodontal Disease. Int. J. Mol. Sci..

[4] Esposito M., Grusovin M.G., Papanikolaou N., Coulthard P., Worthington H.V. (2009). Enamel matrix derivative (Emdogain) for periodontal tissue regeneration in intrabony defects. A Cochrane systematic review. Eur. J. Oral Implantol..

[5] Graziani F., Gennai S., Petrini M., Bettini L., Tonetti M. (2019). Enamel matrix derivative stabilizes blood clot and improves clinical healing in deep pockets after flapless periodontal therapy: A Randomized Clinical Trial. J. Clin. Periodontol..

[6] Graziani F., Peric M., Marhl U., Petrini M., Bettini L., Tonetti M., Gennai S. (2020). Local application of enamel matrix derivative prevents acute systemic inflammation after periodontal regenerative surgery: A randomized controlled clinical trial. J. Clin. Periodontol..

[7] Villa O., Wohlfahrt J.C., Koldsland O.C., Brookes S.J., Lyngstadaas S.P., Aass A.M., Reseland J.E. (2016). EMD in periodontal regenerative surgery modulates cytokine profiles: A randomised controlled clinical trial. Sci. Rep..

[8] Sato S., Kitagawa M., Sakamoto K., Iizuka S., Kudo Y., Ogawa I., Miyauchi M., Chu E.Y., Foster B.L., Somerman M.J. (2008). Enamel matrix derivative exhibits anti-inflammatory properties in monocytes. J. Periodontol..

[9] Myhre A.E., Lyngstadaas S.P., Dahle M.K., Stuestol J.F., Foster S.J., Thiemermann C., Lilleaasen P., Wang J.E., Aasen A.O. (2006). Anti-inflammatory properties of enamel matrix derivative in human blood. J. Periodontal Res..

[10] Ramenzoni L.L., Annasohn L., Miron R.J., Attin T., Schmidlin P.R. (2021). Combination of enamel matrix derivative and hyaluronic acid inhibits lipopolysaccharide-induced inflammatory response on human epithelial and bone cells. Clin. Oral Investig..

[11] Almqvist S., Werthén M., Lyngstadaas S.P., Gretzer C., Thomsen P. (2012). Amelogenins modulate cytokine expression in LPS-challenged cultured human macrophages. Cytokine.

[12] Stout B.M., Alent B.J., Pedalino P., Holbrook R., Gluhak-Heinrich J., Cui Y., Harris M.A., Gemperli A.C., Cochran D.L., Deas D.E. (2014). Enamel Matrix Derivative: Protein Components and Osteoinductive Properties. J. Periodontol..

[13] Gruber R., Bosshardt D.D., Miron R.J., Gemperli A.C., Buser D., Sculean A. (2013). Enamel matrix derivative inhibits adipocyte differentiation of 3T3-L1 cells via activation of TGF-βRI kinase activity. PLoS ONE.

[14] Gruber R., Stähli A., Miron R.J., Bosshardt D.D., Sculean A. (2015). Common target genes of palatal and gingival fibroblasts for EMD: The microarray approach. J. Periodontal Res..

[15] Stephanopoulos G., Garefalaki M.E., Lyroudia K. (2005). Genes and Related Proteins Involved in Amelogenesis Imperfecta. J. Dent. Res..

[16] Hu J.C., Sun X., Zhang C., Simmer J.P. (2001). A comparison of enamelin and amelogenin expression in developing mouse molars. Eur. J. Oral Sci..

[17] Fong C.D., Hammarström L. (2000). Expression of amelin and amelogenin in epithelial root sheath remnants of fully formed rat molars. Oral Surg. Oral Med. Oral Pathol. Oral Radiol. Endod..

[18] Yamamichi K., Fukuda T., Sanui T., Toyoda K., Tanaka U., Nakao Y., Yotsumoto K., Yamato H., Taketomi T., Uchiumi T. (2017). Amelogenin induces M2 macrophage polarisation via PGE2/cAMP signalling pathway. Arch. Oral Biol..

[19] Stähli A., Bosshardt D., Sculean A., Gruber R. (2014). Emdogain-regulated gene expression in palatal fibroblasts requires TGF-βRI kinase signaling. PLoS ONE.

[20] Gruber R., Roos G., Caballé-Serrano J., Miron R., Bosshardt D.D., Sculean A. (2014). TGF-βRI kinase activity mediates Emdogain-stimulated in vitro osteoclastogenesis. Clin. Oral Investig..

[21] Hausmann E.H., Hao S.Y., Pace J.L., Parmely M.J. (1994). Transforming growth factor beta 1 and gamma interferon provide opposing signals to lipopolysaccharide-activated mouse macrophages. Infect. Immun..

[22] Xiao Y.Q., Freire-de-Lima C.G., Janssen W.J., Morimoto K., Lyu D., Bratton D.L., Henson P.M. (2006). Oxidants selectively reverse TGF-beta suppression of proinflammatory mediator production. J. Immunol..

[23] Takaki H., Minoda Y., Koga K., Takaesu G., Yoshimura A., Kobayashi T. (2006). TGF-beta1 suppresses IFN-gamma-induced NO production in macrophages by suppressing STAT1 activation and accelerating iNOS protein degradation. Genes Cells.

[24] Reddy S.T., Gilbert R.S., Xie W., Luner S., Herschman H.R. (1994). TGF-beta 1 inhibits both endotoxin-induced prostaglandin synthesis and expression of the TIS10/prostaglandin synthase 2 gene in murine macrophages. J. Leukoc Biol..

[25] Nasirzade J., Kargarpour Z., Hasannia S., Strauss F.J., Gruber R. (2020). Platelet-rich fibrin elicits an anti-inflammatory response in macrophages in vitro. J. Periodontol..

[26] Pourgonabadi S., Müller H.D., Mendes J.R., Gruber R. (2017). Saliva initiates the formation of pro-inflammatory macrophages in vitro. Arch. Oral Biol..

[27] Müller H.D.H.D., Cvikl B.B., Lussi A.A., Gruber R.R. (2016). Salivary pellets induce a pro-inflammatory response involving the TLR4-NF-kB pathway in gingival fibroblasts. BMC Oral Health.

[28] van Caam A., Madej W., Garcia de Vinuesa A., Goumans M.J., Ten Dijke P., Blaney Davidson E., van der Kraan P. (2017). TGFβ1-induced SMAD2/3 and SMAD1/5 phosphorylation are both ALK5-kinase-dependent in primary chondrocytes and mediated by TAK1 kinase activity. Arthritis Res. Ther..

[29] Hammarström L., Heijl L., Gestrelius S. (1997). Periodontal regeneration in a buccal dehiscence model in monkeys after application of enamel matrix proteins. J. Clin. Periodontol..

[30] Heng N.H.M., N’Guessan P.D., Kleber B.M., Bernimoulin J.P., Pischon N. (2007). Enamel matrix derivative induces connective tissue growth factor expression in human osteoblastic cells. J. Periodontol..

[31] Haruyama N., Hatakeyama J., Moriyama K., Kulkarni A.B. (2011). Amelogenins: Multi-Functional Enamel Matrix Proteins and Their Binding Partners. J. Oral Biosci..

[32] Taylor A.L., Haze-Filderman A., Blumenfeld A., Shay B., Dafni L., Rosenfeld E., YoavLeiser Y., Fermon E., Gruenbaum-Cohen Y., Deutsch D. (2006). High yield of biologically active recombinant human amelogenin using the baculovirus expression system. Protein Expr. Purif..

[33] Kawase T., Okuda K., Momose M., Kato Y., Yoshie H., Burns D.M. (2001). Enamel matrix derivative (EMDOGAIN) rapidly stimulates phosphorylation of the MAP kinase family and nuclear accumulation of smad2 in both oral epithelial and fibroblastic human cells. J. Periodontal Res..

[34] Miron R.J., Bosshardt D.D., Zhang Y., Buser D., Sculean A. (2013). Gene array of primary human osteoblasts exposed to enamel matrix derivative in combination with a natural bone mineral. Clin. Oral Investig..

[35] Kawase T., Okuda K., Yoshie H., Burns D.M. (2002). Anti-TGF-beta antibody blocks enamel matrix derivative-induced upregulation of p21WAF1/cip1 and prevents its inhibition of human oral epithelial cell proliferation. J. Periodontal Res..

[36] Okubo K., Kobayashi M., Takiguchi T., Takada T., Ohazama A., Okamatsu Y., Hasegawa K. (2003). Participation of endogenous IGF-I and TGF-beta 1 with enamel matrix derivative-stimulated cell growth in human periodontal ligament cells. J. Periodontal Res..

[37] Panahipour L., Kochergina E., Kreissl A., Haiden N., Gruber R. (2019). Milk modulates macrophage polarization in vitro. Cytokine X.

[38] Panahipour L., Tabatabaei A.A., Gruber R. (2020). Hypoallergenic infant formula lacks transforming growth factor beta activity and has a lower anti-inflammatory activity than regular infant formula. J. Dairy Sci..

[39] Panahipour L., Kochergina E., Laggner M., Zimmermann M., Mildner M., Ankersmit H.J., Gruber R. (2020). Role for Lipids Secreted by Irradiated Peripheral Blood Mononuclear Cells in Inflammatory Resolution in Vitro. Int. J. Mol. Sci..

[40] Fujishiro N., Anan H., Hamachi T., Maeda K. (2008). The role of macrophages in the periodontal regeneration using Emdogain gel. J. Periodontal Res..

[41] Okuda K., Miyazaki A., Momose M., Murata M., Nomura T., Kubota T., Wolff L.F., Yoshie H. (2001). Levels of tissue inhibitor of metalloproteinases-1 and matrix metalloproteinases-1 and -8 in gingival crevicular fluid following treatment with enamel matrix derivative (EMDOGAIN). J. Periodontal Res..

