# Supplementary material for: TGF-β Signalling Mediates the Anti-Inflammatory Activity of Enamel Matrix Derivative In Vitro
Source: Int J Mol Sci. 2022 Aug 29;23(17):9778. doi: 10.3390/ijms23179778 (PMC9456059; doi:10.3390/ijms23179778)

Supplement figures

Figure S1: Dose-response of EMD concentrations on the viability (MTT test) of RAW 264.7 macrophages.

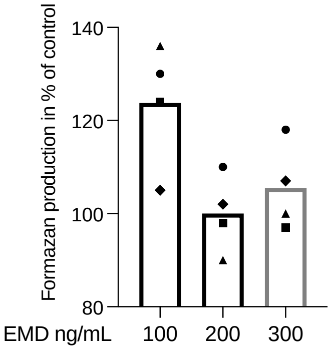

Supplement: Supplementary file 1 [file ijms-23-09778-s001.zip › ijms-1883894-supplementary.pdf]
